# Examining the interplay between *Streptococcus agalactiae,* the biopolymer chitin and its derivative

**DOI:** 10.1002/mbo3.733

**Published:** 2018-10-01

**Authors:** Mediha Yildirim‐Aksoy, Benjamin H. Beck, Dunhua Zhang

**Affiliations:** ^1^ Aquatic Animal Health Research Unit United States Department of Agriculture, Agricultural Research Service Auburn Alabama

**Keywords:** aquaculture, chitin, chitosan, chitosanase, diseases, *Streptococcus*

## Abstract

*Streptococcus agalactiae* is a highly pathogenic bacterium of aquatic species and terrestrial animals worldwide, whereas chitin and its derivative chitosan are among the most abundant biopolymers found in nature, including the aquatic milieu. The present investigation focused on the capability of *S. agalactiae* to degrade and utilize these polymers. Growth of *S. agalactiae* in the presence of colloid chitin, chitosan, or N‐acetyl‐glucosamine (GlcNAc) was evaluated. Chitosanase production was measured daily over 7 days of growth period and degraded products were evaluated with thin later chorography. Chitin had no effect on the growth of *S. agalactiae*. Degraded chitin, however, stimulated the growth of *S. agalactiae*. *S. agalactiae* cells did not produce chitinase to degrade chitin; however, they readily utilize GlcNAc (product of degraded chitin) as sole source of carbon and nitrogen for growth. Chitosan at high concentrations had antibacterial activities against *S. agalactiae*, while in the presence of lower than the inhibitory level of chitosan in the medium, *S. agalactiae* secrets chitosanase to degrade chitosan, and utilizes it to a limited extent to benefit growth. The interaction of *S. agalactiae* with chitin hydrolytes and chitosan could play a role in the diverse habitat distribution and pathogenicity of *S. agalactiae* worldwide.

## 1 INTRODUCTION

Streptococcosis is caused by gram‐positive, globally distributed bacteria capable of infecting more than 27 species of fish inhabiting fresh, brackish, and marine waters (Bowater et al., 2012; Chou et al., 2014; Keirstead et al., 2014; Klesius, Shoemaker, & Evans, 2008; Sheehan et al., 2009). *Streptococcus agalactiae* in particular not only causes invasive disease in fish but also poses a zoonotic hazard. Some strains of aquatic S. agalactiae have potential for pathogen transmission between aquatic animals and humans (Delannoy et al., 2013). *S. agalactiae*‐induced streptococcosis causes substantial fish mortalities and large economic losses on aquaculture operations worldwide, particularly in farmed tilapia (*Oreochromis* sp.), rainbow trout (*Oncorhynchus mykiss*), grouper (*Epinephelus lanceolatus*), and hybrid striped bass (*Morone chrysops* X *M. saxatilis*) (Shoemaker, Xu, Garcia, & LaFrentz, 2017; Shoemaker, Xu, & Soto, 2017). Outbreaks of streptococcosis have also been described in wild fish, e.g., in mullet in Kuwait bay (Evans et al., 2008; Jafar et al., 2008) and in giant Queensland grouper and other wild fish in Australia (Bowater et al., 2012). Other aquatic or semiaquatic species such as marine mammals (Evans et al., 2008; Zappulli et al., 2005), crocodiles (Bishop et al., 2007), bullfrogs (Amborski, Snider, Thune, & Culley, 1983), and terrestrial mammals such as cattle, dogs, cats (Brochet et al., 2006; Sørensen, Poulsen, Ghezzo, Margarit, & Kilian, 2010), and humans were reportedly susceptible to *S. agalactiae* infection. In humans, *S. agalactiae* was found to colonize the rectovaginal tract and cause neonatal infectious disease (Manning et al., 2009; Phares et al., 2008) and may also cause meningitis, septicemia, as well as localized infections (Chaiwarith et al., 2011). The drivers behind the emergence of *S. agalactiae* disease in humans and other animals are still poorly understood.

As an autochthonous bacterium of diverse aquatic ecosystems, the interaction between *S. agalactiae* and chitin is of interest; particularly as chitin is one of the most abundant biopolymers in aquatic environments. Indeed, each year more than one billion tons of chitin is produced in the biosphere by many organisms, mainly by insects, fungi, crustaceans, and other marine and freshwater organisms (Gooday, 1990). Chitin consists of unbranched and degradable chains of *β* (1,4)‐linked N‐acetyl‐glucosamine (GlcNAc) residues and is part of the cell wall structure in invertebrates, protozoa, fungi, and certain groups of algae (Flach, Pilet, & Jolles, 1992). Relatedly, chitosan is a collective name for a group of partially or fully deacetylated chitin, although the degree of the N‐deacetylation is almost never complete (Rabea et al., 2003). In nature, the polymer is partially acetylated and, in fact, the name chitosan describes a wide range of polymers with randomly distributed various proportions of D‐glucosamine (GlcN) (deacetylated unit) and N‐acetyl‐D‐glucosamine (acetylated unit) residues (Pelletier & Sygusch, 1990). Chitosan is also found in the cell wall of several fungi (Pochanavanich & Suntornsuk, 2002), in the green algae *Chlorella* sp. (Mihara, 1961), yeast, protozoa, and in insect cuticles (Aruchami, Gowri, & Sundara‐Rajulu, 1986).

Among bacteria, chitin also provides necessary carbon and nitrogen sources for energy metabolism. Except for *Serratia marcescens*, which is one of the best‐studied chitinolytic bacteria (Vaaje‐Kolstad, Horn, Sørlie, & Eijsink, 2013), many aquatic bacteria such as *Vibrio* sp. can live solely on chitin (Rathore & Gupta, 2015). Recently, a hypervirulent fish strain of *Aeromonas hydrophila* was found to use chitin and chitosan (partially deacetylated chitin) as sole carbon sources for growth (Zhang, Xu, Qiu, & Beck, 2017). Accordingly, the enormous amounts of chitin/chitosan production and continuous deposition of these highly insoluble polymers in the biosphere are degraded by chitinase and chitosanase (GIA, 2010). Chitinase (EC 3.2.1.14) and chitosanase (EC 3.2.1.132) are two members of the glycoside hydrolase family and are characterized by their ability to catalyze the hydrolytic cleavage of chitin and chitosan, respectively (Dahiya, Tewari, & Hoondal, 2006). Chitinase catalyzes the degradation of chitin and these enzymes detected in a wide variety of organisms including bacteria, fungi, viruses, plants and insects (Gooday, 1990; Henrissat & Davies, 1997; Keyhani & Roseman, 1997; Saito et al., 1999). Chitosanase or GlcN‐ase is a hydrolytic enzyme acting on β‐1,4‐glycosidic linkage of chitosan to release chito‐oligosaccharides (Liu et al., 2009). Several microorganisms including actinomycetes, fungi, and bacteria as well as some plants have been reported to produce chitosanase to degrade chitosan and were able to use chitosan as a sole carbon source (Lee et al., 1996; Shimosaka, Nogawa, Wang, Kumehara, & Okazaki, 1995) including *Sphingomonas* sp. (Zhu, Zhou, & Feng, 2007), and *Bacillus* sp. (Choi, Kim, Piao, Yun, & Shin, 2004; Kurakake, Yo‐u, Nakagawa, Sugihara, & Komaki, 2000; Zakaria, Zakaria, Musa, Hamilin, & Zulkifly, 2012). The chitin‐degrading enzyme (chitinase) in *Yersinia entomophaga* functions as an integral part of toxin complex (Busby et al., 2012). For *V. cholerae*, its association with chitin was related with its chemotaxis, cell multiplication, induction of competence, biofilm formation, as well as pathogenicity for humans and aquatic animals (Pruzzo, Vezzulli, & Colwell, 2008).

Because of the diverse habitat distribution of *S. agalactiae* and global abundance of chitin/chitosan as a major component of organic matter in aquatic ecosystems, elucidating their interactions will augment our understanding of these bacteria and potentially guide the development of preventatives and therapeutic approaches for disease control. Therefore, the purpose of this study was to examine the *in vitro* interactions of *S. agalactiae* with chitin, chitosan, and their hydrolysis products to reveal new clues aimed at minimizing disease outbreaks of these globally important bacterial pathogens.

## 2 MATERIALS AND METHODS

### 2.1 Bacterial strain and culture conditions methods

The strain, TN‐Hon‐084 of *Streptococcus agalactiae* (Shoemaker, Xu, et al., 2017; Shoemaker, Xu, & Soto, 2017) originally isolated from diseased fish, was used in this study. The bacterium was routinely cultured and maintained in tryptic soy broth (TSB) (Fisher Scientific, Pittsburgh, PA) at 28°C.

### 2.2 Colloidal chitin and chitosan preparation

Colloidal chitin was prepared according to the method of Murthy and Bleakley (2012) with some modifications. Briefly, 1 g chitin powder from shrimp shells (Sigma–Aldrich, St. Louis, MO) was dissolved in 15 ml concentrated hydrochloric acid (HCl) and vigorously stirred for 1 hr in a chemical fume hood at room temperature. Chitin was precipitated as a colloidal suspension by adding 250 ml distilled water. Aliquots of the chitin suspension were dispensed in 50 ml tubes and centrifuged at 4000 rpm for 10 min. Precipitates were washed 3 ×  with distilled water. The pH was adjusted to 5.5 with NaOH. The concentration of colloidal chitin was adjusted to 30 mg/ml. The preparation was then autoclaved at 121°C for 15 min and stored at 4°C until use.

Chitosan (CS) was purchased from Sigma–Aldrich (St. Louis, MO). Chitosan stock solution was prepared by dissolving in 0.2 N HCl at a concentration of 1% (w/v) with stirring overnight at 30°C. The pH of the solution was then adjusted to 5.5 with NaOH. Stock solution of 0.2N HCl (chitosan solvent) was prepared and pH was adjusted to 5.5 to determine the effect of HCl. Stock solutions were sterilized at 121°C for 15 min in an autoclave and stored at 4°C after cooling.

### 2.3 Effect of colloid chitin on growth of *Streptococcus agalactiae*

The effect of colloid chitin on the growth of *S. agalactiae* and the ability of *S. agalactiae* to use colloid chitin as a sole carbon source to grow were assessed using TSB and M9 minimal medium (MM) solution supplemented with 0.6% colloid chitin. The MM contained 47.8 mM Na_2_HPO_4_, 22.0 mM KH_2_PO_4_,8.5 mM NaCl,18.7 mM NH_4_Cl, 20 mM MgSO_4_ and 0.1 mM CaCl_2_. To prepare working solution, 20 ml 5× minimal salts, 400 μl of 1M MgSO_4_ and 200 μl of 0.1M CaCl_2_ was added and made up to 100 ml. Stock solutions were sterilized at 121°C for 15 min in an autoclave. Growth media (TSB or MM) without colloid chitin served as controls. Approximately 2 × 10^8^ colony forming units (CFU)/ml of *S. agalactiae* were washed with MM, and inoculated to 50 ml of growth medium, resulting in the start cell density of 4 × 10^7^ CFU/ml. The culture was kept at 28°C with constant shaking at 130 rpm. An aliquot of 100 μl of culture was sampled daily for 4 days and numbers of CFU were determined by conventional plate count method using tryptic soy agar (TSA) as growth medium.

### 2.4 Preliminary chitinase activity assay

Extracellular chitinolytic activity of *S. agalactiae* was evaluated by applying cell culture on agar medium containing colloidal chitin. Aliquots of 10 μl of the bacterial culture were spotted on the surface of MM agar containing 0.2% colloid chitin. Two plates were used, with each plate having three spots. Plates were incubated at 28°C until halo zones (clearing of opaque medium) formed around the colonies. *Aeromonas hydrophila,* ML‐10‐51K, which is known for having chitinolytic activity, was used as a positive control (Zhang, Bland, Xu, & Chung, 2015; Zhang et al., 2017).

### 2.5 Growth of *bacteria* in M9 minimal medium containing N‐acetyl‐D‐glucosamine (GlcNAc) or chitosan (CS) as sole carbon sources

Kinetic growth was assayed by measuring the turbidity of a cell suspension in 96‐well microtiter plates. Bacteria were grown in M9 minimal media, supplemented with various concentrations of GlcNAc or CS, and no carbon source served as control. Overnight culture of bacterial cells was centrifuged and resuspended in M9 minimal medium, and the optical density was adjusted to OD_540_ = 0.3. Aliquots of 125 μl bacterial suspension were added to each well of a 96‐well plate. Then, 250 μl GlcNAc or CS with concentrations varied from 0.125 to 4% and from 0.003 to 0.1%, respectively, was added to each well. Wells without addition of GlcNAc or CS served as controls. There were quadruplicate replicate wells for each concentration. Kinetic of bacterial growth in various concentrations of GlcNAc or CS were measured at 28°C with constant shaking for 30 and 72 hr, respectively, using an Epoch‐2 microplate reader (BioTek, Winooski, TX, USA).

### 2.6 Coculture of *Streptococcus agalactiae* with an extracellular chitinase‐producing aquatic bacterium

To determine if *S. agalactiae* was capable of scavenging chitin degraded products produced by other chitinolytic bacterium, *S. agalactiae* was cocultured with *Aeromonas hydrophila* ML‐10‐51K. Overnight cultures of *S. agalactiae* and *A. hydrophila* were washed with MM medium and inoculated individually or together in MM medium (final volume of 2.5 ml) containing 0.6% colloid chitin. Disappearance of colloidal chitin from the test tubes was daily inspected for 5 days. Samples were centrifuged at 3000 rpm for 10 min and remaining levels of colloid chitin in test tubes were photographed.

To obtain extracellular chitinolytic enzyme, *A. hydrophila* was cultured in M9 minimal medium in 50 ml tubes containing 0.6% colloid chitin (after washing once with MM) until all the chitin disappeared. Cell‐free culture supernatant was obtained by centrifugation at 5000 rpm for 15 min and filtered through a 0.22 μm Millipore^®^ filter. The filter‐sterilized supernatant was divided to two parts and one part was heated at 100°C for 30 min to inactivate chitinolytic enzymes. The cell‐free supernatant with active or inactivated chitinase and minimal medium (2.5 ml) were then supplemented with 0.6% colloid chitin. M9 minimal medium without colloid chitin served as control. Cells of *S. agalactiae* washed with MM medium, were inoculated with starting cell density of approximately 3 × 10^7^ CFU/ml. MM medium supplemented with same level of colloid chitin was also inoculated with *S. agalactiae*. The culture was kept at 28°C with constant shaking at 130 rpm. An aliquot of 100 μl of culture was sampled daily for 4 days and numbers of CFU were determined using the same method described above.

### 2.7 Effect of chitosan on bacterial cell proliferation

Cell proliferation assays were conducted by measuring the turbidity of cell suspensions in 96‐well microtiter plates. The bacterial culture (OD_540_ = 1.0) were serially diluted at 1 to 1,000 ratio in TSB. A total volume of 125 μl bacterial suspension in triplicate was added to each well (except first row) of a 96‐well plate. A total volume of 250 μl chitosan or HCl at a concentration of 0.8% in bacterial suspensions was added to first row. Serial twofold dilutions of chitosan or HCl made in bacterial suspension to give final chitosan concentrations of 0.8, 0.4, 0.2, 0.1, 0.05, 0.025, and 0% (w/v). Negative controls (chitosan + TSB or HCl + TSB) were also assayed in triplicate. Kinetics of bacterial growth were measured at 28°C with constant shaking for 24 hr using an Epoch‐2 microplate reader (BioTek, Winooski, TX). Absorbance values of negative controls were subtracted from the corresponding sample absorbance readings. After incubation, subsamples from each concentration were strike in TSB agar plates containing no CS or HCl to test bacterial recovery (i.e., to determine whether viable cells were present). The samples were re‐incubated at 28°C for 24–72 hr and the growth of bacterial colonies was observed.

### 2.8 Bacterial cell viability

The amounts of viable bacteria in each well were determined using an assay of MTT (3‐(4,5‐dimethylthiazol‐2‐yl)‐2,5‐diphenyl‐2*H*‐tetrazolium bromide (Sigma‐Aldrich (St. Louis, MO). A stock MTT solution (5 mg/ml) in phosphate‐buffered saline (PBS), pH 7.4 was prepared immediately prior to use, and filter‐sterilized with a 0.22 μm Millipore^®^ filter. Three sets of bacterial culture in 96‐well microtiter plates (one for each time course, 24, 48, and 72 hr incubation), identical to above, were prepared and incubated at 28°C for 20, 44, and 68 hr. The MTT stock solution was then added to each well at a final concentration of 0.5 mg/ml, and the mixture was incubated at 28°C for 4 hr. The formazan produced was solubilized by addition of 125 μl acidified isopropanol (0.04N HCl in isopropanol). The quantity of dissolved formazan (presumably directly proportional to the number of viable cells) was measured at 570 nm using an Epoch‐2 microplate reader.

### 2.9 Culture conditions for production of chitosanase

To produce chitosanase, the pre‐grown bacteria were inoculated in 35 ml M9 minimal medium containing 0.05% chitosan and grown in an orbital shaking incubator (130 rpm) for 7 days at 28°C. Daily, cell viability was estimated by using MTT assay as described above and 4 ml of subsamples centrifuged and resulting supernatants were saved at −20°C until use for measuring enzyme activities.

### 2.10 Assessment of chitinase and chitosanase activities in extracellular products (ECP)

Chitinase activity was measured with colloidal chitin as a substrate by measuring the amount of the reducing end group, NAG, degraded from colloidal chitin, as described by Imoto and Yagishita (1971) with some modifications. The chitosanase activities were determined by measuring the amount of N‐acetyl‐D‐glucosamine (GlcNAc) or glucosamine (GlcN) degraded from chitin and chitosan, respectively. Briefly, corresponding crude enzymes in ECP (0.9 ml) were mixed at 3:1 ratio with 2% colloidal chitin or 1% chitosan. After incubation for 2 hr at 37°C for chitinase and 56°C for chitosanase, the reaction mixture was subjected to refrigerated centrifugation (4°C) at 13,000 rpm for 10 min. The resulting supernatant (0.7 ml) was mixed with 70 μl of 0.1% NBT solution and boiled for 20 min. The NBT solution was made by dissolving 0.1 g NBT in 50 ml 0.05M NaOH and mixing with equal volume of 0.5 M sodium potassium tartrate. (Both solutions were filtered through a 0.22 μm Millipore^®^ filter before mixing). After cooling, the absorbance of the mixture was measured at 660 nm using an Epoch‐2 microplate reader. The activity was calculated from a standard curve obtained using NAG or glucosamine. One unit (U) of the enzyme (chitinase or chitosanase) activity was defined as the amount of enzyme that yield 1 μmol of reducing sugar as N‐acetyl‐D‐glucosamine (GlcNAc) or glucosamine (GlcN) equivalent per minute.

### 2.11 Thin layer chromatography (TLC)

Chitosan hydrolysis was qualitatively analyzed by means of TLC using silica gel plate (DC Kieselgel 60, Merck KGaA). ECP‐mediated chitosanase enzyme was obtained from the cultures described above in the culture conditions for production of chitosanase by centrifugation at 5000 rpm for 30 min. The resulting supernatant (3 ml) was filtered through a 0.22 m filter (PES membrane) unit (Millex‐GP, Merck Millipore Ltd, Tullagreen, IRL) to remove remaining bacterial cells and concentrated to a volume of approximately 0.5 ml using a Pierce Concentrator (PES, 20k MWCO; Thermo Scientific, Rockford, IL). The concentrated supernatant (0.5 ml) was mixed at 3:1 ratio with 1% chitosan. After incubation for 24 hr at 37°C, the reaction mixture was subjected to refrigerated centrifugation at 13,000 rpm for 10 min.

TLC was performed to analyze ECP‐mediated chitosanase enzyme using modified method of Bond, Tsai, and Russell (1999). Briefly, an aliquot of 2–6 μl of samples was spotted on a TLC plate. Chromatography was developed in a solvent containing ethyl acetate:butanol:acetic acid:water (80:10:5:5, v/v) for 90 min. The plate was allowed to air‐dried and stained by spraying with *p*‐anisaldehyde reagent (*p*‐anisaldehyde: ethanol:sulfuric acid:acetic acid; 1:18:0.5:0.5, v/v) and heated at 120°C for 5 min.

### 2.12 Statistical analysis

Graphs and standard error of means (SEM) of the data were performed using Graphpad Prism 6.0 (San Jose, CA, USA).

## 3 RESULTS

**Figure 1:**
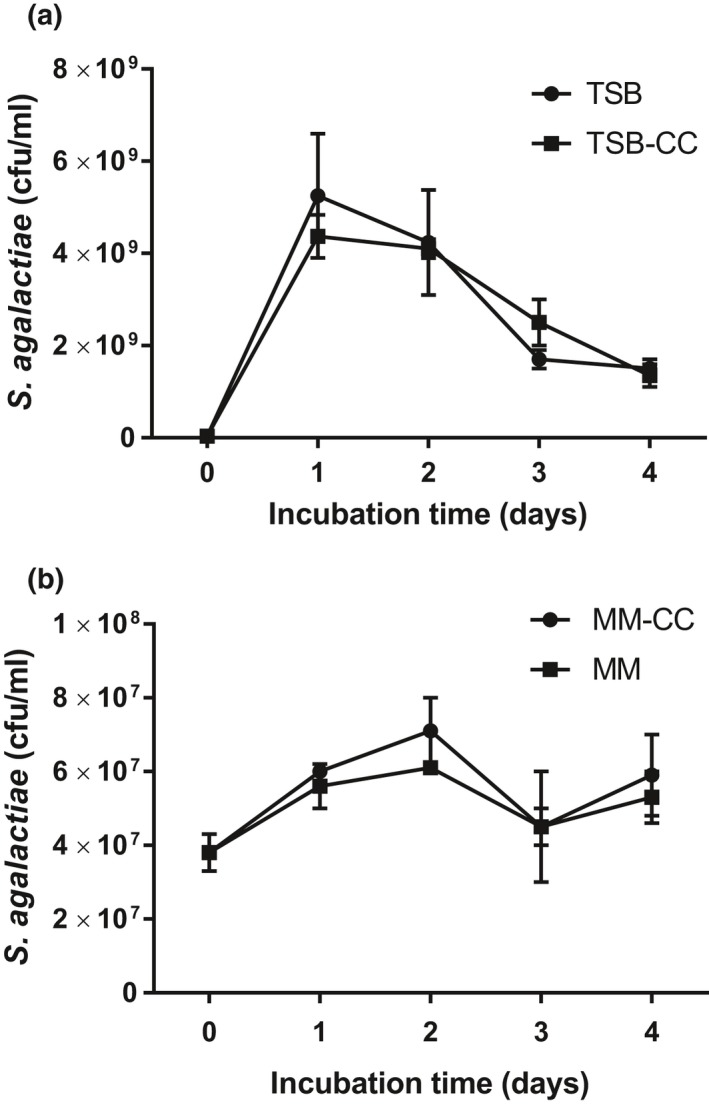
Effect of colloid chitin (CC) on growth of *Streptococcus agalactiae* TN‐Hon‐084 in growth medium (tryptic soy broth, TSB) (a) or M9 minimal medium (MM) (b) for 4 days. Error bars represent standard error of means (SEM) from three replicates

Colloidal chitin had no influence on the growth of *Streptococcus agalactiae* in either TSB (Figure 1a) or M9 minimal medium (Figure 1b). Preliminary tests showed that clear hydrolysis zones formed around *Aeromonas hydrophila* ML‐10‐51K colonies, which was used as a positive control, on colloidal chitin agar. However, *S. agalactiae* did not form such hydrolytic zones. We also analyzed chitinase enzyme activities in cell‐free culture supernatants of *S. agalactiae* incubated with colloid chitin medium. Similarly, *S. agalactiae* did not excrete detectable levels of chitinase to hydrolyze colloid chitin (data are not shown).

**Figure 2:**
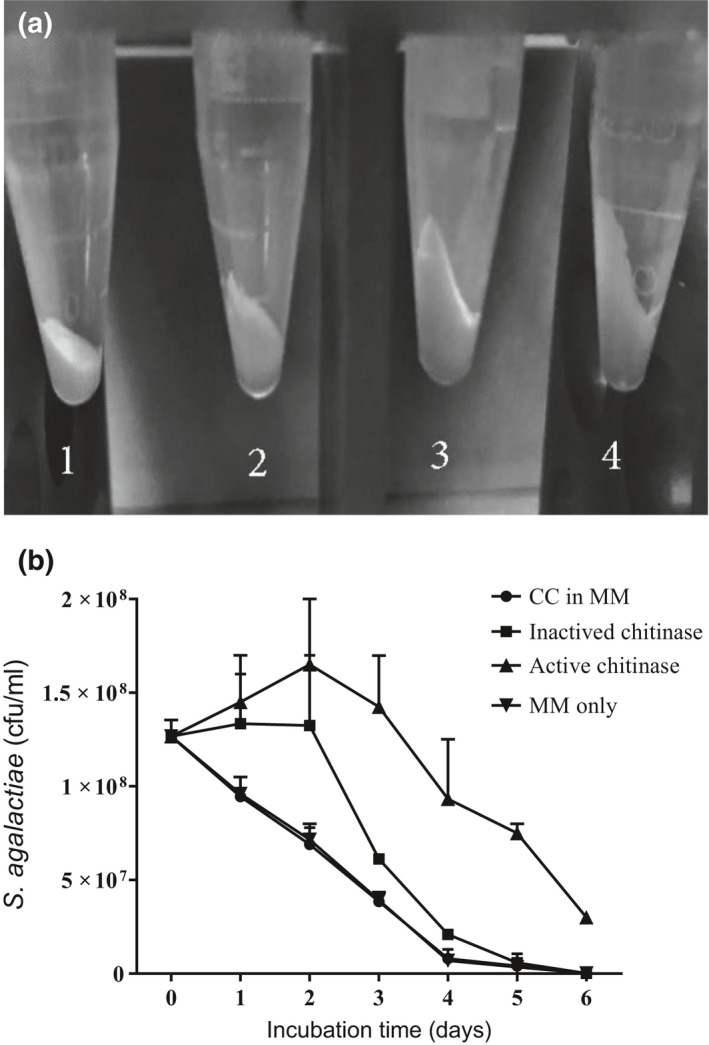
Amount of colloid chitin left after culturing *Streptococcus agalactiae* TN‐Hon‐084 with or without *Aeromonas hydrophila* ML‐10‐51K in minimal medium for 10 days. Tube 1—Coculture of *S. agalactiae* and *A. hydrophila* and; Tube 2—*A. hydrophila* alone; Tube 3— *S. agalactiae* alone; Tube 4—Colloid chitin alone (a). Influence of cell‐free culture supernatant of *A. hydrophila* ML‐10‐51K containing chitinolytic enzyme on growth of *S. agalactiae* TN‐Hon‐084 in M9 minimal medium (MM) containing colloid chitin (CC) for 6 days (b). Error bars represent standard error of means (SEM) from three replicates

Even though, they did not produce chitinolytic enzymes to hydrolyze chitin, we next cocultured *S. agalactiae* with a chitinase‐producing bacterium (*A. hydrophila* ML‐10‐51K) to test if *S. agalactiae* can coexist and scavenge degraded products produced by other chitinolytic bacteria. Apparently, *S. agalactiae* was able to grow in the coculture even though it could not degrade colloid chitin itself (Figure 2a). Colloid chitin disappeared slightly faster in coculture compared to sole culture of chitinase‐producing bacterium. In the cell‐free culture supernatant of *A. hydrophila* grown in medium containing colloid chitin as a sole carbon source, the number of *S. agalactiae* cells increased up to 2 days (CFU increased from 1.3x10^8^ to 1.6x10^8^) and then, constantly decreased over the next 4 days (Figure 2b). The number of cells remained constant for 2 days in the cell‐free culture supernatant containing inactivated chitinolytic enzyme suggesting presence of degraded chitin. There was a linear decrease in the number of colony counts in the control groups.

**Figure 3:**
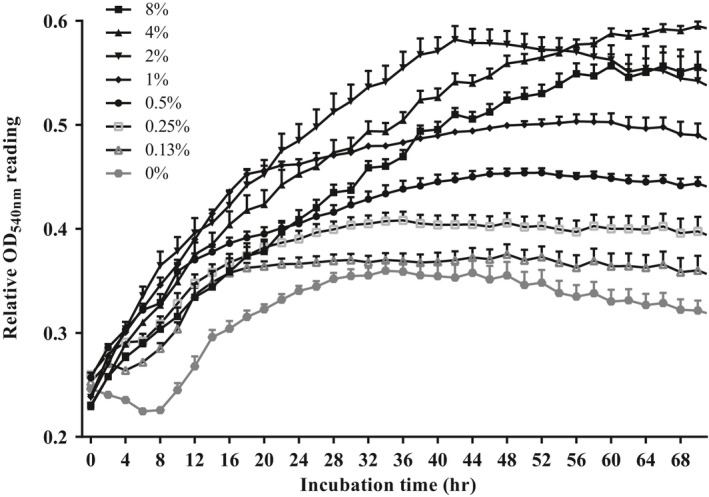
Kinetic growth of *Streptococcus agalactiae* TN‐Hon‐084 in M9 minimal medium containing various concentrations of N‐acetyl‐D‐glucosamine (GlcNAc) as a sole carbon source. Relative cell growth determined by the turbidity measured at OD_540 nm_. Data are presented as mean ± standard deviation (*SD*) from three replicates

The growth curve in M9 minimal media containing various concentrations of GlcNAc as a sole carbon source was conducted to test if they can utilize the monomeric unit of the polymer chitin (Figure 3). Log phase was longer in bacteria with higher levels of GlcNAc in the medium. *S. agalactiae* incubated in medium containing 1% and 2% GlcNAc reached to stationary phase after 16 and 40 hr of incubation, respectively. *S. agalactiae* cultured in medium containing 4% and 8% GlcNAc was still on log phase at 72 hr of incubation. At the end of the 72‐hr incubation period, absorbance reading was increasingly higher with increasing concentration of GlcNAc in the culture medium up to 4%.

**Figure 4:**
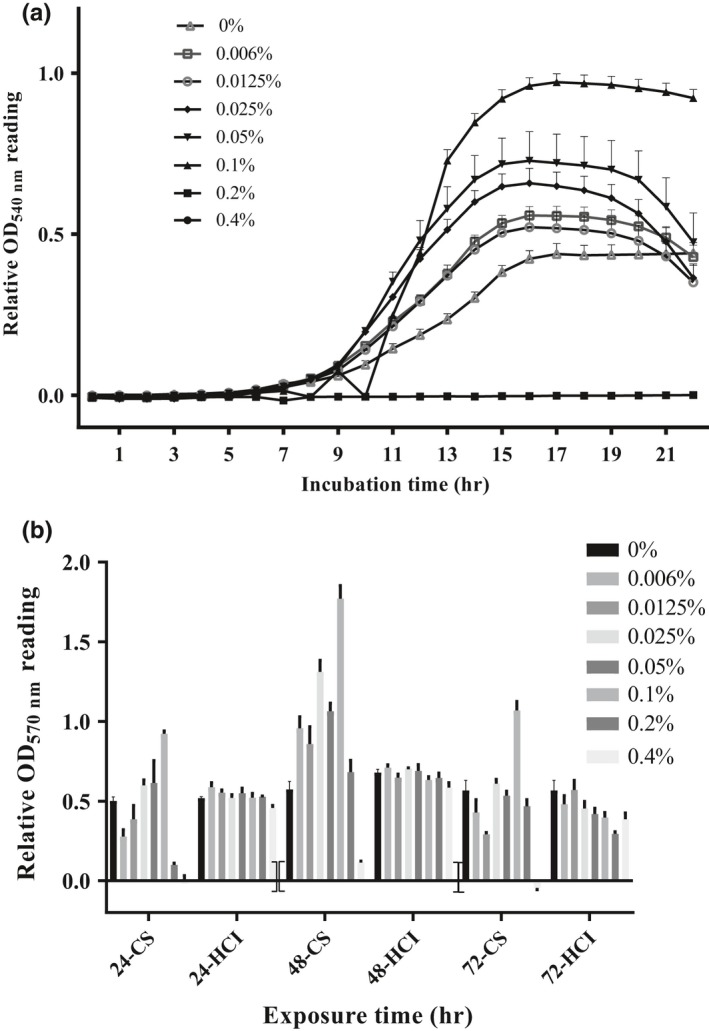
The kinetic growth (a) and relative cell viability (survival) (b) of *Streptococcus agalactiae* TN‐Hon‐084 incubated with various concentrations of chitosan. Relative cell growth determined for 24 hr with 1‐hr interval by the turbidity measured at OD_540 nm_. Cell viability was estimated by MTT assay and absorbance was measured at OD_570 nm_ in 96‐well plates following 24‐, 48‐, or 72‐hr exposure to chitosan. Data are presented as mean ± standard deviation (SD) from three replicates. All experiments were performed duplicate

Chitosan exhibited antibacterial activity at the concentration of 0.2% or higher against *S. agalactiae* (Figure 4a,b). On the other hand, absorbance reading of chitosan‐treated groups at the concentrations of 0.1% and lower was higher than that of the control group (Figure 4a). It completely inhibited growth of *S. agalactiae* at the concentration of 0.4% (w/v) over a 72‐hr incubation period based on MTT assay. Chitosan at a concentration of 0.1% had the highest reading regardless of the exposure time. In the medium containing 0.2% chitosan, growth of both bacteria was strongly inhibited up to 24‐hr incubation period. After 48‐hr incubation, however, there were no differences in the absorbance values of *S. agalactiae* between chitosan‐treated groups (0.2%, w/v) and untreated controls. The effect of HCl as a chitosan solvent was also evaluated at the same concentrations used in chitosan‐treated groups on the relative survival at 24, 48, and 72 hr with *S. agalactiae*. No antibacterial activity of HCI was observed against on growth and survival of bacterium (Figure 4b).

**Figure 5:**
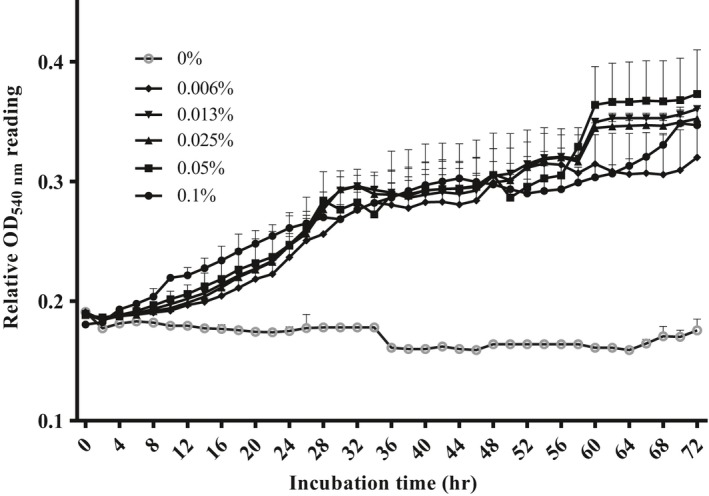
Kinetic growth of *Streptococcus agalactiae* TN‐Hon‐084 in M9 minimal medium containing various concentrations of chitosan as a sole carbon source. Relative cell growth determined by the turbidity measured at OD_540 nm_. Data are presented as mean ± standard deviation (*SD*) from three replicates

To monitor chitosan utilization by *S. agalactiae* as a sole carbon source, growth curves in minimal medium containing gradient levels of chitosan were determined (Figure 5). Absorbance readings of all chitosan‐treated groups from 0.006% to 0.1% were markedly higher than that of the control group. Absorbance reading of control group remained unchanged throughout the incubation period. At the end of the 72‐hr incubation period, cell densities in medium containing chitosan concentration from 0.125% to 0.1% were similar and reached stationary phase at 60 hr of incubation.

**Figure 6:**
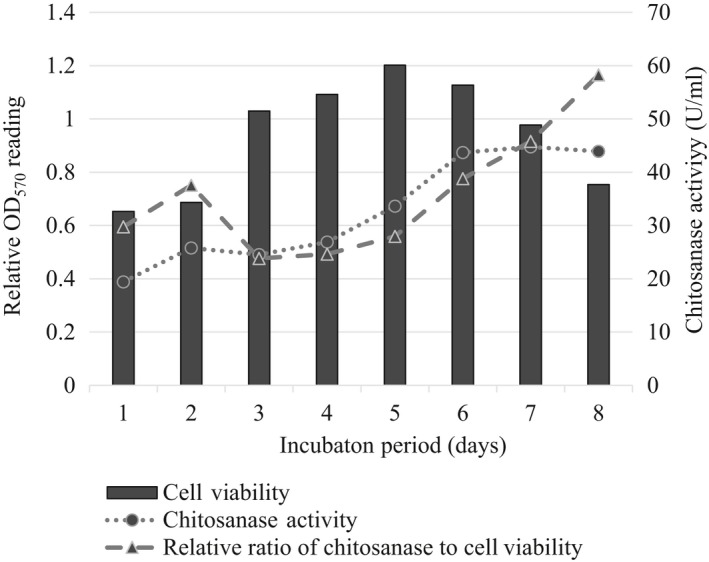
Chitosanase activity compared to relative cell viability (survival) of *Streptococcus agalactiae* TN‐Hon‐084 incubated in liquid M9 minimal medium 0.05% chitosan at 28°C for 8 days. Data are presented as mean ± standard deviation (*SD*) from three replicates

Time course of chitosanase productions by *S. agalactiae* correlated with the viable bacterial cells as shown in Figure 6. Cultivation of *S. agalactiae* cells in a M9 medium supplemented with 0.05% chitosan as a sole carbon source excreted chitosanase into the culture medium. The level of extracellular chitosanase produced by *S. agalactiae* and the amount of viable bacterial cells increased with increasing the incubation time up to day 6 and day 5, respectively. This point further, the level of chitosanase in the medium remained unchanged in contrast to decreasing amount of surviving bacterial cells. The low growth of bacteria at day 2 in the medium containing chitosan was due to the inhibitory activity of chitosan on bacterial growth. After day 3, constant increase of the relative ratio of chitosanase to amount of live bacteria in the medium was observed throughout the cultivation period.

**Figure 7:**
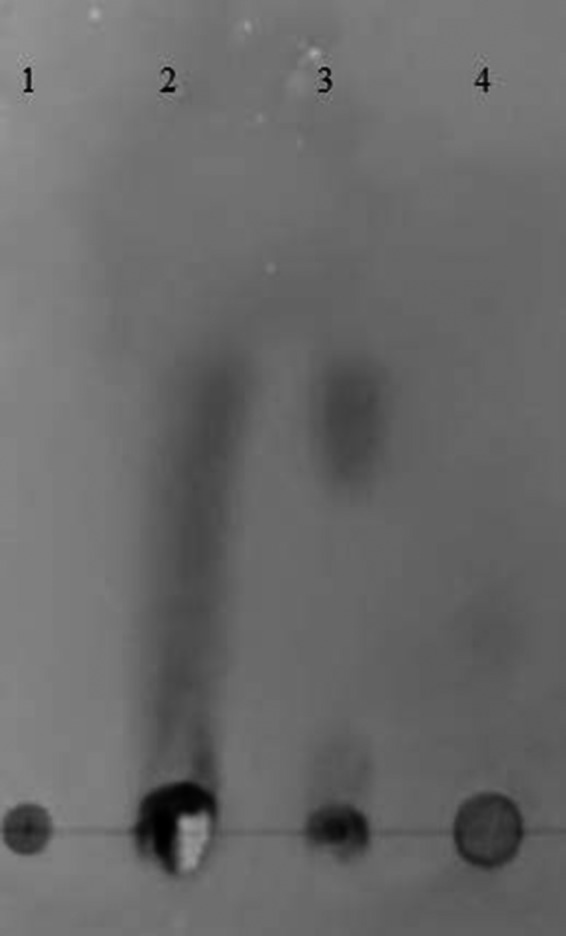
Thin layer chromatograph (TLC) of chitosan‐degrading products by extracellular products (ECP) *of Stroptococcus agalactiae* TN‐Hon‐084. Lane 1. Chitosan alone (not treated with ESP); Lane 2. Chitosan treated with ECP from *S. agalactiae*; Lane 3. ECP from *S. agalactiae* cultured in chitosan containing medium; and Lane 4. ECP from *S. agalactiae* cultured in TSB without chitosan

The hydrolytic ability of extracellular products (ECP) from *S. agalactiae* was analyzed using thin layer chromatograph (TLC) (Figure 7). When ECP from stimulated *S. agalactiae* for chitosanase production was incubated with chitosan as substrate for 2 hr at 37°C, various lengths of chitosan oligosaccharides were liberated from chitosan. On the other hand, no detectable degradation products were observed in ECP of unstimulated bacteria during growth (lane 4). ECP from S*. agalactiae* grown in minimal medium in the presence of chitosan to stimulate chitosanase production for 8 days alone revealed remaining of shorter chained oligosaccharide from cleavage of chitosan. No degradation product was detected in control (chitosan without ECP (lane 1).

## 4 DISCUSSION

Chitin and chitosan are abundant biomaterials. Chitin is the second most abundant polymers on earth after cellulose and possibly the most abundant in the aquatic environment. Although chitosan is commercially produced from shrimp and crab shell by deacetylation of chitin with strong alkalis at high temperatures for long periods of time (Knorr, 1991), it is also found in nature including the cell walls of several fungi (Pochanavanich & Suntornsuk, 2002), in the green algae *Chlorella* sp. (Mihara, 1961), and in yeast, protozoa, and insect cuticles (Aruchami et al., 1986). In aquatic environments, available chitosan may provide *S. agalactiae* with sources of nutrients and energy. From the present results, there was no evidence that *S. agalactiae* can degrade chitin. However, it thrived on degraded chitin products without investing energy in the biosynthesis of this enzyme. During growth, chitin disappeared faster from medium containing both bacterial species than that containing only the chitin‐degrading strain (*A. hydrophila*). It was assumed that cells with no apparent chitinase activity fed on hydrolytic products generated in excess by the chitinase‐positive populations. This type of multicellular cooperation could be a strategy observed in bacterial communities (Shapiro, 1998) and has been described for several chitinolytic strains (Chernin et al., 1998; DeAngelis, Lindow, & Firestone, 2008; Gaffney et al., 1994).

Several culture independent studies in aquatic environments that quantify the fraction of chitin degraders vs. chitin consumers in the total bacterial community support the existence of significant cross feeding during chitin degradation: chitinolytic organisms were estimated to represent average about 1% of all prokaryotes in a variety of aquatic ecosystems (Beier, Jones, Mohit, Hallin, & Bertilsson, 2011; Cottrell, Moore, & Kirchman, 1999). An even lower fraction of cells displayed active chitinolytic activity in natural aquatic habitats (Beier & Bertilsson, 2011; Beier et al., 2012). *S. agalactiae* is incapable of degrading chitin but can utilize N‐acetyloglucosamine as the sole source of carbon and nitrogen. Mobley, Doyle, Streips, and Langemeier (1982) found that when GlcNAc was used as a sole carbon source, N‐acetylglucosamine sugar was utilized in cell macromolecular synthesis and energy metabolism; However, if GlcNAc did not serve as the sole source of carbon, then ~90% of GlcNAc taken up into the cells was in turn incorporated into the cell walls in the *Bacillus subtilis* (Mobley et al., 1982). The ability to take up GlcNAc appears to be widespread in aquatic bacteria (Beier & Bertilsson, 2011; Eckert, Baumgartner, Huber, & Pernthaler, 2013; Nedoma, Vrba, Hejzlar, Šimek, & Straškrabová, 1994; Riemann & Azam, 2002).

The antibacterial activity of chitosan and its derivatives has been recognized against bacteria, (Benhabiles et al., 2012; Kong, Chen, Xing, & Park, 2010; Liu, Du, Wang, & Sun, 2004; No, Park, Lee, & Meyers, 2002; Sudarshan, Hoover, & Knorr, 1992; Wei & Xia, 2003; Xia, 2003; Yang, Chou, & Li, 2005; Zheng & Zhu, 2003), fungal, and/or viral pathogens (Hirano & Nagao, 1989; Uchida, Lzume, & Ohtakara, 1989). Similar activities have been reported for food bacteria, mold, and yeast (Benhabiles et al., 2012; No et al., 2002; Wei & Xia, 2003). Recently, we demonstrated that chitosan and its derivatives inhibited the growth of three pathogenic gram‐negative bacteria of warm water finfish (Yildirim‐Aksoy & Beck, 2017). In the present study, the antibacterial activities of chitin and chitosan were also examined against *S. agalactiae*. Colloidal chitin had no effect on growth of the *S. agalactiae*; however, at concentrations of 0.2% or higher, chitosan inhibited the growth of this bacterium. By examining relative antibacterial activities of chitin, chitosan, and its oligomers against four gram‐positive and seven gram‐negative bacteria by Benhabiles et al. (2012), they found that chitosan exhibited a bacteriostatic effect on the gram‐negative bacteria, *Escherichia coli* ATCC 25922, *Vibrio cholerae, Shigella dysenteriae*, and *Bacteroides fragilis* but not against *Salmonella typhimurium*. The antibacterial mechanism of chitosan is mainly acting on the outer surface of bacteria by binding to the oppositely charged bacterial surface to cause agglutination. Its interaction with the membrane of the cell alters cell permeability causing leakage of cytoplasmic components and finally death of the cell (Yildirim‐Aksoy & Beck, 2017). A probable explanation for the stimulated growth with the low chitosan‐treated groups is that there was insufficient polysaccharide to interact and kill all the bacteria in the culture medium and the amino groups of chitosan were bound to cell debris or surface components of the bacteria and were no longer available to attach to other cell surfaces. Therefore, surviving *S. agalactiae* cells continued reproducing and possessed the ability to degrade and utilize chitosan to benefit their growth. Several microorganisms including actinomycetes, fungi, and bacteria have been reported to produce chitosanase to degrade chitosan and use it as a sole carbon source (Lee et al., 1996; Shimosaka et al., 1995). Chitosan‐degrading bacteria have been identified in *Sphingomonas* sp. (Zhu et al., 2007) and *Bacillus* sp. (Choi et al., 2004; Kurakake et al., 2000; Zakaria et al., 2012).

Even though, being not capable of producing chitinase to degrade chitin, *S. agalactiae* secretes chitosanase into surrounding medium in response to chitosan induction. The distinction between chitinases and chitosanases is that chitinase specifically cleaves the *N*‐acetyl‐D‐glucosaminidic bonds while chitosanase cleaves the β‐D‐glucosaminidic bonds. Chitosanases can hydrolyze all kinds of linkages in chitosan except for the GlcNAc‐GlcNAc bond (Zhu, Wu, & Dai, 2003). However, some chitinases hydrolyze GlcN‐GlcNAc bonds in addition to GlcNAc‐GlcNAc ones. Apparently, *S. agalactiae* required chitosan to induce the production of chitosanase (Figure 7). While some microorganisms constitutively produced chitosanase without chitosan, most microorganisms needed chitosan as an inducer to produce chitosanase (Kurakake et al., 2000; Lee et al., 1996; Shimosaka et al., 1995). In this study, *S. agalactiae* was investigated for secretion of chitosanase during its growth. The changes of chitosanase in the culture supernatant were analyzed for 8 days in chitosan medium and correlated with the viable bacterial cells. It showed that *S. agalactiae* produced extracellular chitosanase continuously and increased steadily during the exponential phase of growth when growth medium containing 0.05% chitosan as substrate. The highest level of chitosanase was found on the sixth day of culture when the bacterium entered the late stationary phase, then it remains unchanged although cell count was gradually declined. This result may be due to the accumulation of chitosanase in the medium during previous growth phase. The low growth of bacteria at second day in the medium containing chitosan may be due to the inhibitory activity of chitosan on bacterial growth.

In gram‐positive bacteria, peptidoglycan makes up as much as 90% of the thick cell wall enclosing the plasma membrane. The peptidoglycan layer in the bacterial cell wall is formed from linear chains of two alternating amino sugars, namely N‐acetylglucosamine (GlcNAc) and N‐acetylmuramic acid (MurNAc). Chitosan is a linear polysaccharide composed of randomly distributed β‐(1→4)‐linked D‐glucosamine (deacetylated unit) and N‐acetyl‐D‐glucosamine (acetylated unit). Chitin and chitosan are distinguished by the amount of acetylation of the D‐glucosamine (GlcN) residues. *S. agalctiae*, a gram‐positive bacterium, might be using N‐acetyl‐glucosamine (GlcNAc) residues of chitin and acetylated units of chitosan to build the peptidoglycan layer in their cell walls. The bacterium was able to decompose chitosan and utilize D‐glucosamine as additional source of carbon to grow when cells are grown in the growth media (TSB) containing less than inhibitor levels of chitosan. When chitosan was used as the sole carbon source, however, the bacterium can utilize it to a certain extent. Increasing chitosan level did not further stimulate growth of cells. This might be explained by limited levels of acetyl groups in chitosan compared to GlcNAc. This relates to the fact that polymers containing more than 70% acetylating are considered chitin, while those with <30% are called chitosan.

In conclusion, *S. agalactiae* did not possess the ability to degrade chitin but readily utilized degraded chitin products for growth. The bacterium utilized GlcNAc as the only source of carbon and nitrogen. Colloidal chitin has no effect on growth of *S. agalactiae*, however, chitosan at concentrations of 0.2% or higher, inhibited the growth of *S. agalactiae*. Lower inhibitory level of chitosan, on the other hand, stimulates the growth of the bacterium. In the presence of chitosan in the medium, *S. agalactiae* secreted chitosanase to degrade chitosan, and utilized to limited extent to benefit its growth. The interaction of *S. agalactiae* with chitin hydrolytes and chitosan plays a role in diverse habitat distribution of *S. agalactiae* worldwide.

## CONFLICT OF INTEREST

All authors certify that there is no conflict of interest with any financial/research/academic organization. Mention of trade names or commercial products in this article is solely for the purpose of providing specific information and does not imply recommendation or endorsement by the U.S. Department of Agriculture.

## AUTHOR CONTRIBUTION

Yildirim‐Aksoy designed and carried out the experiment and wrote the manuscript with support from Beck and Zhang. All authors discussed the results and commented on the manuscript.

## ETHICAL APPROVAL

Not applicable.

## Data Availability

All data generated or analyzed during this study are included in this published article.
